# Copper transporter 1 (CTR1) expression by mouse testicular germ cells, but not Sertoli cells, is essential for functional spermatogenesis

**DOI:** 10.1371/journal.pone.0215522

**Published:** 2019-04-19

**Authors:** Rashin Ghaffari, Kristin R. Di Bona, Christopher L. Riley, John H. Richburg

**Affiliations:** 1 Institute of Cellular and Molecular Biology, College of Natural Sciences, The University of Texas at Austin, Austin, TX, United States of America; 2 The Center for Molecular Carcinogenesis and Toxicology, Division of Pharmacology & Toxicology, College of Pharmacy, The University of Texas at Austin, Austin, TX, United States of America; University of Hyderabad, INDIA

## Abstract

An imbalance in copper (Cu) tissue homeostasis has a degenerative effect on spermatogenesis and male fertility. The high-affinity Cu transporter 1 (CTR1; SLC31A1) is the major protein responsible for Cu acquisition in eukaryotes and is highly expressed in mouse testes. Studies on yeast and *Drosophila* have demonstrated the conserved essential function of Cu and CTR1 for meiosis and fertility, implying that CTR1 may play an essential function in mammalian spermatogenesis. In mice, spermatogenesis takes place within the seminiferous epithelium, where tight junctions between somatic Sertoli cells (SCs) create a specialized microenvironment for the development of meiotic germ cells (GCs) by tightly regulating the free transport of metabolites and ions to reach these cells. Here, it is demonstrated that within the seminiferous epithelium, CTR1 is expressed on the membrane of primary pachytene spermatocytes and SCs. To examine the physiological significance of CTR1 in spermatogenesis, mice with a GC-specific (*Ctr1*^*ΔGC*^) and SC-specific (*Ctr1*^*ΔSC*^) disruption of the *Ctr1* gene were generated. The testis of *Ctr1*^*ΔGC*^ mice exhibits a severe progressive loss of GCs starting at postnatal day (PND) 28 leading to testis hypoplasia by adulthood. No spermatogenic recovery was observed in *Ctr1*^*ΔGC*^ testis beyond PND 41, despite the presence of FOXO-1 expressing undifferentiated spermatogonial cells. However, *Ctr1*^*ΔSC*^ mice displayed functional spermatogenesis and were fertile, even though testicular Cu levels and Cu-dependent cellular activities were significantly reduced. These results reveal, for the first time, the importance of CTR1 expression by GCs for maintaining functional spermatogenesis.

## Introduction

Copper (Cu) is an essential trace metal that is required for all organisms due to its important roles in growth and development. Cu serves as an important co-factor for enzymes that carryout fundamental biological processes including respiration (cytochrome c oxidase), elimination of free radicals (superoxide dismutase), iron metabolism (ceruloplasmin), connective tissue formation (lysyl oxidase) and many others [1,2]. On the other hand, excess Cu can create a toxic environment in the host cell by producing reactive oxygen species [3,4]. Consequently, alterations of Cu levels and the activities of Cu-dependent enzymes lead to disease and pathophysiological conditions including Wilson’s and Menke’s disease, and ataxia [1,3]. The importance of Cu in spermatogenesis has been documented in various animal studies [5]. Induced Cu deficiencies in male rats, goat and rams result in reduced sperm counts and motility, poor semen quality and an abnormal germinal epithelium [6–8]. These effects were reversible upon Cu supplementation indicating the importance of Cu in the maintenance of male fertility and spermatogenesis [6–8].

Spermatogenesis is a complex process in which spermatogonial stem cells (SSCs) proliferate and their progeny (spermatogonia) undergo many successive mitotic divisions ending with the development of meiotic cells (spermatocytes). Spermatocytes subsequently undergo two meiotic divisions leading to the production of haploid cells (spermatids) that further differentiate to give rise to spermatozoa. This process takes place in the seminiferous epithelium of the mammalian testis and is largely orchestrated by the somatic Sertoli cells (SCs) [9]. Specialized tight junctions between adjacent SCs creates a barrier known as blood-testis barrier (BTB) that creates two compartments: a basal compartment below the BTB and adluminal above. The basal compartment is mitotic spermatogonial cells reside and the adluminal compartment is where meiotic spermatocytes, spermatids and spermatozoa are found. The BTB regulates the free transport of metabolites, ions or harmful substances from entering lumen of the seminiferous tubules and reaching the meiotic germ cells (GCs) [10]. Many different transporters are expressed in testis, either by the SCs or GCs, to facilitate the influx and/or efflux of metabolites and create a suitable environment for spermatogenesis to take place [11].

Organisms regulate Cu homeostasis via various proteins responsible for regulating Cu transport, intracellular trafficking and storage [2,12]. The Cu transporter 1 (SLC31A1; CTR1) is a high affinity Cu transporter, conserved from yeast to humans, which functions as a major Cu importer across the plasma membrane [13,14]. The loss of *Ctr1* is embryonically lethal in mice, further confirming its essential function in developmental physiology [15]. The *Ctr1* gene is broadly expressed in all tissues of the mouse however, the relative tissue distribution varies with liver, kidney, and testis expressing higher mRNA levels, while brain and muscle express lower levels [15–18].

There is a growing body of evidence on the effects of Cu and *Ctr1* expression on meiosis and male fertility in eukaryotes. In the *Schizossacchromyces pombe Ctr4*, the yeast homolog of mammalian *Ctr1*, is highly expressed on the cell surface during early meiosis by which cells undergo meiotic arrest at metaphase I in a Cu deficient environment [19,20]. Furthermore, in *Drosophila* the CTR1-like protein, *Ctr1C*, also shown to be associated with male fertility [21]. In *Drosophila* endogenous *Ctr1C* is expressed in mature spermatocytes and spermatozoa while an alternate transcript, *Ctr1B* led expressed in the intestine. Lack of *Ctr1C* in mutant background lacking intestinal *Ctr1B*, lead to male sterility in drosophila [21]. A recent report evaluating the expression profile of Cu regulatory proteins in the mouse testis indicated that the CTR1 protein is mainly observed in the adluminal compartment of the seminiferous epithelium where primary spermatocytes reside [22]. Together, these observations strongly implicate C*tr1* as an important factor for mammalian spermatogenesis. In this study the physiological significance of *Ctr1* in both GCs and in SCs is independently characterized for its importance in functional spermatogenesis.

## Materials and methods

### Generation of germ cell- and Sertoli cell- specific *Ctr1* knockout mice

The GC and SC specific *Ctr1* knockout mice were generated using the *Cre-loxp* system. The transgenic mice strains *Ddx4-Cre* (GC-specific Cre strain number 006954, also known as *Vasa-Cre* [23]) and *Amh-Cre* (SC-specific Cre Stock number 007915[24]) were purchased from The Jackson Laboratory (Bar Harbor, ME). The *Ctr1*^*fl/+*^ mice were generously provided by Dr. Dennis Thiele (Duke University, NC [25]) and maintained in breeding colonies throughout experiments. Mice colonies were kept in a controlled temperature (23°C ± 1°C) and light (12 L:12 D) environment. Standard chow (5LL2, Purina Mills Lab-Diet, St. Louis, MO) and tap water were supplied *ad libitum*. All procedures were performed in accordance with established guidelines and approval from The University of Texas at Austin’s Institutional Animal Care and Use Committee (Protocol ID: AUP-2015-00198).

Initially, homozygous *Ctr1*^*fl/fl*^ female mice were crossed with male mice carrying either *Ddx4-* or *Amh-Cre* gene, which expresses *Cre* recombinase under the control of *Ddx4* or *Amh* gene promoter, respectively. *Ddx4* expression is activated in early GCs (gonocytes or prospermatogonia) on embryonic (E) days 15–18, and *Amh-Cre* is activated as early as E14.5 in SCs [23,24]. The heterozygous mice carrying either *Ddx4-Cre*; *Ctr1*^*+/fl*^ or *Amh-Cre*; *Ctr1*^*+/fl*^ genotype from the initial cross were then back-crossed to *Ctr1*^*fl/fl*^ female mice to obtain GC (*Ctr1*^*ΔGC*^*)* or SC specific (*Ctr1*^*ΔSC*^*) Ctr1* knockouts (S1A Fig). Wild-type littermates carrying *Ctr1*^*fl/fl*^ gene with no *Cre* gene expression were used as control mice (WT). Genotypic conformation was performed via PCR of genomic DNA with the AccuStart II Mouse Genotyping Kit (95135–500, Quanta BioSciences, Gaithersburg, MD, USA,) using primers specific for the presence of the *Amh-Cre* transgene: 5’- TGG TTT CCC GCA GAA CCT GAA G-3’ (forward); 5’- GAG CCT GTT TTG CAG GTT CAC C-3’ (reverse), *Ddx4-Cre* transgene: 5’-CAG GGT GTT ATA AGC AAT CCC-3’ (forward); 5’-CCT GGA AAA TGC TTC TGT CCG-3’ (reverse) and the *Ctr1*-LoxP gene: 5’-AATGTCCTGGTGCGTCTGAAA-3’ (LoxA838U); 5’-GCAGTAGATAAAAGCCAAGGC-3’ (LoxA1052L) [25] (S1B Fig)

### Tissue collection

Mice at postnatal day (PND) ages 14, 28, 41, 60, 70 were either euthanized by CO_2_ asphyxiation followed by cervical dislocation for histological, protein and RNA assay and for metal measurements purposes. Both testes were rapidly removed, weighed, and either flash frozen in liquid nitrogen and stored at -80°C or immersed in Bouin’s solution (R1121000, RICCA Chemical Company, Arlington, TX).

### Histology and immunohistochemistry

Testes were collected and fixed in Bouin’s solution overnight at room temperature, then washed in lithium saturated 70% ethanol and embedded in paraffin. Paraffin-embedded testis was sectioned to a thickness of 5 μm. Sections were then deparaffinized and rehydrated in a graded series of ethanol solution in order to perform histology and immunohistochemical detection of the primary antibodies.

For morphological analyses, testis sections were stained with periodic acid-Schiff-hematoxylin (PAS-H) and mounted on glass slides according to standard protocols [26].

Immunohistological staining was performed according to established protocols [27] using VectaStain ABC kit (PK6101, Vector Laboratories, Burlington, CA, USA) and 3–3’-diaminobenzidine (DAB) substrate (SK-4100, Vector Laboratories). Testis cross sections were first incubated in 10% normal horse serum blocking buffer (Sigma-Aldrich, St. Louis, MO) for an hour at room temperature. Cross sections were then incubated overnight at 4°C with the specific primary antibody diluted in 10% horse serum blocking buffer, including rabbit polyclonal anti-CTR1 (1:500, 071314/1-2; kindly provided by Dr. Dennis Thiele [25]), and rabbit anti-FOXO-1 (1:100, 2880, Cell Signaling Technologies Inc. Danvers, MA), or incubated for one hour at room temperature with either rabbit anti-PCNA (1:200, ab18197, Abcam, Cambridge, MA), or polyclonal rabbit anti-SOX9 (1:200, AB5535, EMD Millipore, Burlington, MA).

Histological sections were imaged using a Nikon Eclipse microscope and captured using Nikon digital sight DS-Fi1 camera. Images were analyzed using NIS Elements (version 3.2 64 Bit) and ImageJ (version 1.50i) software.

### Assessment of Sertoli cell numbers

The SC marker, SOX9, was used to determine the average number of SCs per tubule for each genotype [27]. The average number of SCs per tubule was calculated by counting SOX9 positively nuclear stained cells in each round seminiferous tubule (> 100 tubules per mouse per genotype). A total of 3 animals per genotype were analyzed.

### TUNEL assay for apoptosis

Terminal deoxynuceotidyl transferase dUTP nick end labeling (TUNEL) staining was used to assess GC apoptosis in paraffin-embedded testis cross sections. TUNEL assay was performed using the ApopTag Peroxidase *In Situ* Apoptosis Detection Kit (S7100, EMD Millipore). The apoptotic index (AI) was calculated as the percentage of essentially round seminiferous tubules cross sections containing > 3 TUNEL-positive GCs [28]. More than 100 seminiferous tubules per cross section were quantified per animal. A total of 3 animals per genotype per age group were analyzed.

### Sertoli cell isolation

The SC isolation procedure was performed as described previously Karzai et al. with minor modifications [29]. Testes of PND 25 mice were collected, detunicated and processed through two rounds of enzymatic digestions (0.1% collagenase, 0.2% hyaluronidase, 0.03% DNaseI, 0.03% Soybean trypsin inhibitor, pH7.4) at 34°C, 80 oscillations/min for 25 minutes each. Cells were then washed with Hanks solution twice, followed by two rounds of enzymatic digestions (0.1% collagenase–dispase, 0.2% hyaluronidase, 0.03% DNaseI, 0.03% Soybean trypsin inhibitor, pH7.4). Cells were washed twice with Hanks solution and then filtered through a 70–100 μm Nitex membrane and the flow through cells were collected and plated. Cells were plated (3-4x10^6^ cells per 60mm plate) in laminin coated 60 mm plates and incubated at 35°C (95% O_2_ and 5% CO_2_). Media was changed every 24 hours to remove unattached GC contaminants. By day 7, SCs cells were either collected for RNA or protein assays. The purity of the SC culture was assessed using *in situ* staining of SOX9 antibody. The procedure yielded approximately 85–90% SC purity.

### Quantitative-PCR

RNA from primary SC cultures was collected using PureLink RNA Mini Kit (12183020, Ambion) according to the manufactures protocol. A total of 500 ng RNA was used to make cDNA. Q-PCR was performed on each cDNA sample of 10 μl volume containing 1 μl of 20 ng cDNA, 1x of iTaq Universal SYBR Green Supermix (1725120, Bio-Rad Laboratories Inc., Hercules, CA) and 250 nM forward and reverse primers for *Ctr1*: 5’-GGGGCTTACCCTGTGAAGACTTT-3’ (forward); 5’-CGTCCGTGTGGTTCATACCC-3’ (reverse). Relative mRNA expression for the gene of interest was normalized against the housekeeping gene Hypoxanthine guanine phosphoribosyl transferase 1 (*hprt1*) [30]: 5’-CAGTCCCAGCTCGTGATTA-3’ (forward); 5’-TGGCCTCCCATCTCCTTCAT -3’ (reverse) using the ΔΔCT method as described in Applied Biosystems User Bulletin No. 2 (P/N 4303859).

### Membrane preparation for CTR1 protein detection

Crude membrane preparations from primary SC pellets were homogenized with homogenizing buffer containing 10 mM Tris-HCL, 1 mM EDTA, 250mM NaCl and Mini Protease Inhibitor (88666, Thermo Scientific, Waltham, MA) and incubated at 4°C for 10 minutes. The cell homogenates were centrifuged at 21,000 g at 4°C for 15 minutes. The supernatant (cytosolic fraction) was removed, and the pellet was re-suspended in homogenizing buffer containing 1% Triton X-100. The solution was then incubated at 4°C for 30 minutes and centrifuged with 21,000 g 4°C for 5 minutes. The supernatant was collected as crude membrane fraction and used for immunoblotting against CTR1 antibody (as described below).

### Immunoblotting

Protein lysates were prepared from mice testis by homogenizing in RIPA buffer containing 1% Triton X-100, 1 mM EDTA, 0.1% SDS; freshly prepared with complete Mini Protease Inhibitor (88666, Thermo Scientific). Protein concentrations were measured using bicinchoninic acid (BCA) assay (23224, 23228, Thermo Scientific) with bovine serum albumin (23210, Thermo Scientific) as standard. A total of 20 μg of protein lysates were ran through SDS-PAGE gel and transferred onto Polyvininylidene Fluoride (PVDF) membrane. The PVDF membrane was first blocked with 5% milk and probed overnight at 4°C with either mouse monoclonal anti-CCS (1:100, sc-374205, Santa Cruz, Dallas, TX), or rabbit polyclonal anti-SOD1 (1:100, NBP2-24915, Novus, Littleton, CO), or rabbit polyclonal anti-COX5A (1:500, C1129A, CusAb), or rabbit anti-GAPDH (1:1000, 5174S, Cell Signaling) all diluted in 2.5% milk.

Western blotting for CTR1 was performed on membrane enriched primary SCs (20 μg) lysates. A rabbit polyclonal anti-CTR1 was generated and affinity purified of the antigen peptide H_2_N- VSIRYNSMPVPGPNGTILC-CO_2_H, which corresponds to the cytosolic loop between transmembrane domains 1 and 2 of mouse and human CTR1 by Bethyl laboratories (Montgomery, TX). The CTR1 antibody (1:1000 dilution) was incubated overnight at 4°C in 2.5% milk. Overnight incubation at 4°C with rabbit polyclonal anti-Sodium/Potassium pump (1:100, SC-28800, Santa Cruz) was used as a plasma membrane protein marker.

Either HRP-conjugated mouse-IgG (1:1000 dilution) or rabbit-IgG (1:2500 dilution) was used as the appropriate secondary antibody for immunoblotting. The protein bands were visualized using ECL reagent (GE healthcare RPN2232) as described by the manufacturer.

### Cytochrome c oxidase activity assay

Cytochrome c oxidase (CCO) activity assay was performed according established protocols [31]. A total of 10 μg of protein lysates from adult testis and 15 μg primary SCs protein lysates of both *Ctr1*^*ΔSC*^ and WT were used. A solution of 100 mM potassium phosphate buffer (pH 7.0) and 1 mM of reduced cytochrome c was first measured for baseline activity at 550 nm for 2 minutes. The enzyme activity was calculated from the decrease rate of absorbance of cytochrome c at 550 nm (ξ = 18.5 nM^-1^ cm^-1^) for 3 minutes following the addition of the samples. Since the estimate of the CCO enzyme activity depends on the amount of mitochondria within the tissue or cell, the CCO activity was normalized to the activity of citrate synthase enzyme, a mitochondrial matrix enzyme used as marker of the abundance of mitochondria. Citrate synthase was assayed by the reduction of 1 mM of 5,5′-Dithiobis (2-nitrobenzoic acid) (DTNB) in the sample containing 200 mM Tris buffer (pH 8.0) with Triton X-100 (0.2% (vol/vol)), 10 mM of acetyl CoA, and 10 mM oxaloacetic acid. The increase in absorbance was monitored at 412 nm for 3 minutes (ξ = 13.6 mM^-1^ cm^-1^). Cells and tissues samples of three animals from each genotype were used for analysis.

### Testicular spermatid head counts

To estimate sperm production from each genotype, testicular spermatid head counts were performed as previously described with a few modifications [32]. Testis from each mouse was homogenized in 10% DMSO/Saline solution. Cell suspension was then centrifuged at 7,500 rpm for 2 minutes. The pellet was re-suspended in DMSO/Saline solution and was diluted 1:1 ratio in 0.1% Trypan-blue stain (15250–016, GIBCO, ThermoFisher,). The sperm suspension was loaded onto a hemocytometer and the number of spermatid heads was counted via light microscopy. Three animals from each genotype were analyzed.

### Total metal measurements

Testes and seminiferous tubules were collected and weighed into acid washed vials (175–54, Savillex) and dried overnight at 85°C. Dried tissues were then digested overnight into 1 ml of trace-analysis grade nitric acid (225711, Sigma) at 85°C. Metal measurements of the primary SCs were performed according to Zogzas et. al. with few modifications [33]. On day 7 of primary SC culture, cells were washed with cold phosphate buffered saline (PBS) and harvested by trypsin and pelleted. Cell pellets were then washed twice with PBS supplemented with 10 mM EDTA. Before the final wash, pellet was re-suspended in EDTA-free PBS and the number of cells were counted using Trypan-blue (T8154, Sigma) using hematocytometer for each pellet per animal. Following the final wash, PBS supernatant were aspirated completely, and pellets were directly digested in 200 μl of trace-analysis grade nitric acid and incubated at 85°C overnight. Both tissue and primary cell samples were brought to room temperature and reduced nitric acid concentration to 2% nitric acid with ultra-pure water (10977–015, life Technologies). Samples were then sent to inductively coupled plasma- mass spectrometry (ICP-MS) core facility in Jackson School of Geosciences, of the University of Texas at Austin for analysis.

### Statistical analyses

Statistical analyses were performed utilizing GraphPad Prism version 7.03 for Windows. Data are presented as the means ± SD and analyzed using unpaired t-tests corrected for multiple comparisons using the Holm-Sidak method. Statistical significance was considered when *p* < 0.05 unless otherwise stated.

## Results

### CTR1 is expressed in pachytene spermatocytes and in Sertoli cells in a stage specific manner

**Fig 1:**
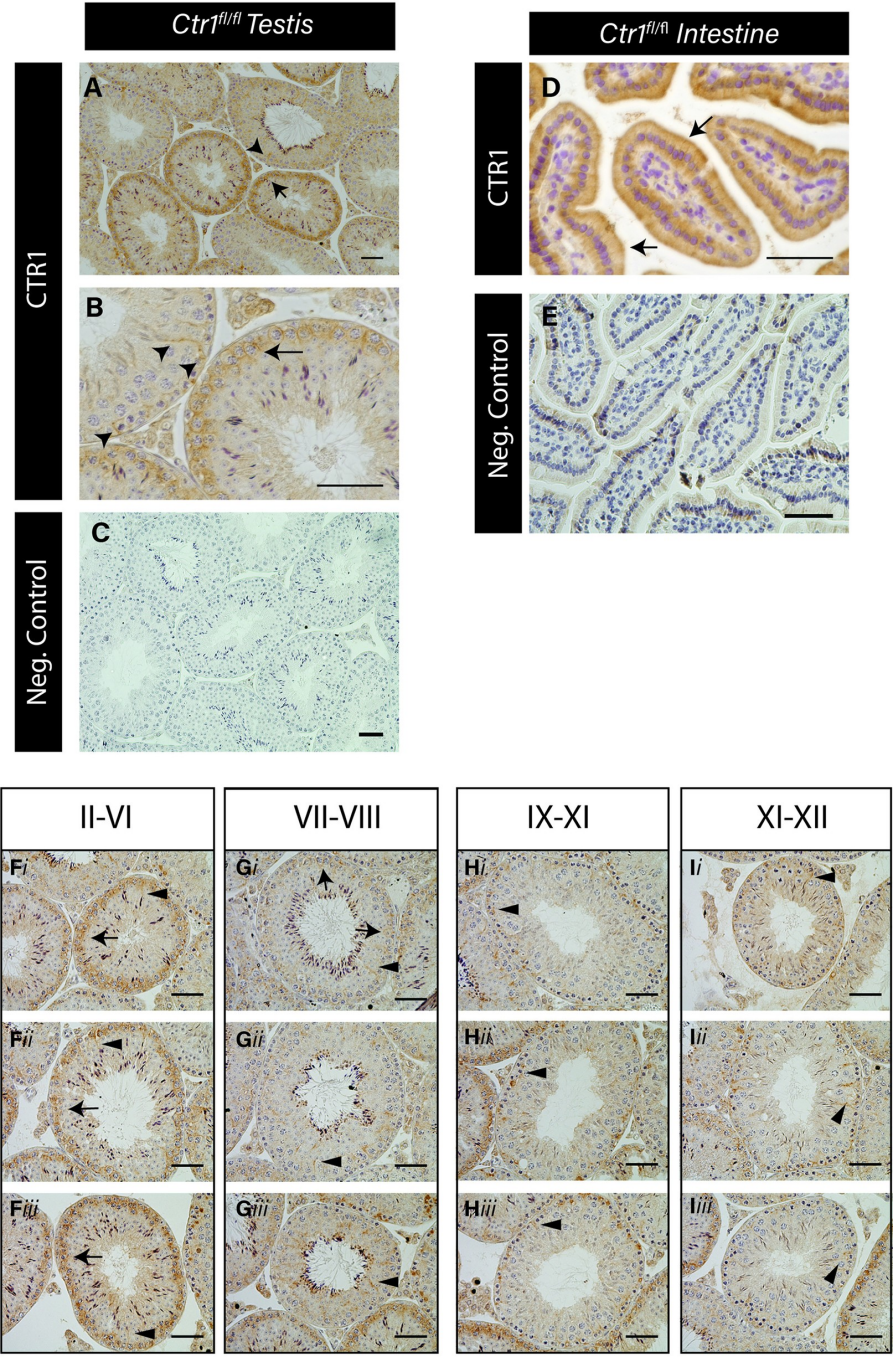
CTR1 is expressed in pachytene spermatocytes and Sertoli cells in a stage specific manner. Immunohistochemical localization of CTR1 in adult (PND 60) *Ctr1*^*fl/fl*^ mouse testis cross sections (A, B). Arrow indicates expression on primary pachytene spermatocytes. Arrowheads indicate SC apical and basal cytoplasm. CTR1 expression on the apical surface of the epithelial cell in the intestine of adult mice indicated by arrows (D). Negative secondary only control of testis (C) and intestine (E) shown. Scale bar = 50 μm. Seminiferous epithelium at stages II-VI displayed the highest CTR1 expression on pachytene spermatocytes (F). Stages VII-XII (G, H and I) display the least CTR1 staining on primary spermatocytes (arrows) but high CTR1 localization on SCs (indicated by arrowheads) within the seminiferous tubule cross sections. Three different tubule cross sections for each specified stage are shown below each grouped of stages (i-iii). Scale bar = 100 μm.

To ascertain the specific cellular localization of CTR1 protein within seminiferous epithelium, immunohistochemical analysis was performed with a previously well-characterized specific antibody of CTR1 that was examined across mammalian species [34]. The localization of CTR1 protein in testicular cross sections of PND 60 wild-type (WT, *Ctr1*^*fl/fl*^) mice testes indicated that within the seminiferous tubules, CTR1 localized to the plasma membrane of primary spermatocytes and along both the basal and adluminal compartments of SCs (Fig 1A and 1B). The specificity of the CTR1 antibody was confirmed on adult intestine cross sections (Fig 1D), which show CTR1 expression on the apical membrane of the intestinal epithelial cells, consistent with previous reports [25,34]. Secondary only negative control is shown in Fig 1C and 1E.

Immunohistochemical analysis on testis cross sections revealed a stage specific pattern of CTR1 protein expression within the seminiferous tubules. In histological cross section of mammalian testis, recurring of multiple GCs at various stages of development are always found associated together within a linear segment of seminiferous tubules. These recurring GC associations goes through defined cycles within seminiferous tubules that are referred to as stages, ranging from I to XII, of spermatogenic cycle in mice (for review, see [9,35]). Histological stages of the seminiferous epithelium in WT mouse testis cross sections were estimated by assessing the shape of spermatid heads, acrosomal cap, presence and location of preleptotene spermatocytes [35]. The stages were accordingly grouped as II-VI, VII-VIII, IX-XI and XII. Fig 1F illustrates that CTR1 expression detected on primary pachytene spermatocytes was highest in tubules at stages II to VI, and in few tubules at stages VII-VIII (Fig 1G). CTR1-expressing SCs were also evident in stages II-VIII (Fig 1F and 1G). However, within stages IX-XII, CTR1 staining was mostly evident on SCs and not on GCs (Fig 1H and 1I). Together, these results show that CTR1 is expressed principally by SCs and the spermatocytes with the highest expression of CTR1 found in the primary pachytene spermatocytes within seminiferous tubule stages II-VI. CTR1 stage specific expression pattern in the testes of C57BL/6J mice was also evident as shown in S1C Fig.

### *Ctr1*^*ΔGC*^ mice exhibit severe loss of germ cells with increasing age

**Fig 2:**
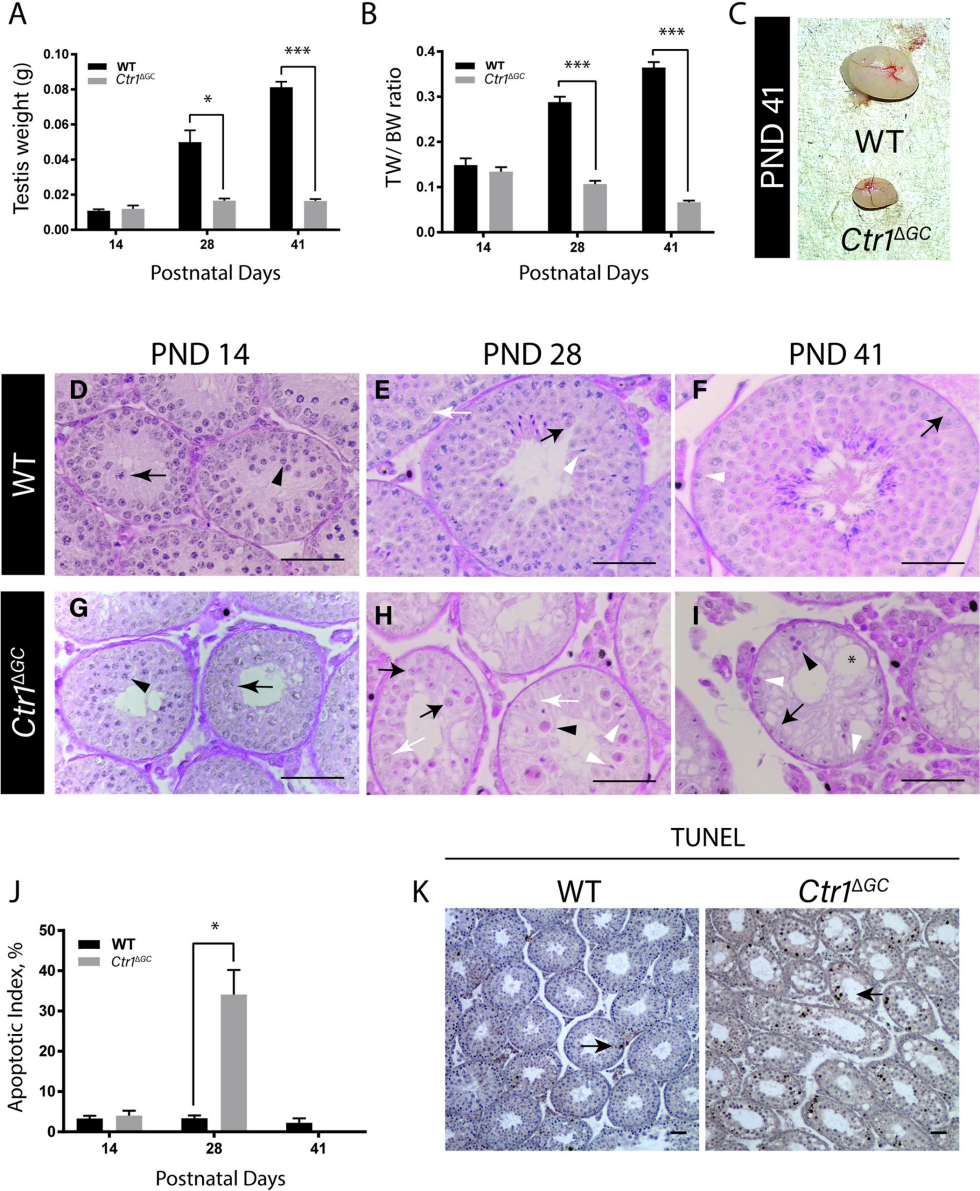
*Ctr1*^*Δ*GC^ mice exhibit severe loss of germ cell with increasing age. Total testis weight (A), and testis to body weight (TW/BW) ratios (B) of WT and *Ctr1*^*ΔGC*^ mice over time with a representative image of PND 41 testis (C) of both WT and *Ctr1*^*ΔGC*^ mice. (N = 3 for each age; *p <0.05 and *** p < 0.001). Histological cross sections of WT (D, E and F) and *Ctr1*^*Δ*GC^ (G, H and I) seminiferous tubules on PND 14 (left), PND 28 (middle), and PND 41 (right) with PAS-H staining. Scale bar = 50 μm. *Ctr1*^*Δ*GC^ testis at PND 14 (D and G) arrowhead indicating zygotene spermatocytes and arrow indicating pachytene spermatocytes. *Ctr1*^*Δ*GC^ testis at PND 28 (E and H) black arrow indicates round spermatids; white arrow indicates pachytene spermatocytes; black arrowhead indicates apoptotic GCs; white arrowhead indicates elongated spermatids. *Ctr1*^*Δ*GC^ testis at PND 41 (F and I) white arrowhead indicates residual pre-meiotic (preleptotene and spermatogonia) GCs; arrow indicates SCs; black arrowhead indicates apoptotic cells; star sign indicates SC vacuole. (J) Apoptotic index in WT or *Ctr1*^*ΔGC*^ on each PNDs (N = 3 for each age; *p < 0.05). (K) TUNEL staining indicating apoptotic cells (arrow) in the seminiferous tubules of PND 28 WT or *Ctr1*^*ΔGC*^ mice at peak apoptosis. Scale = 100 μm.

To examine the functional significance of CTR1 in testicular GCs, mice with a specific disruption of the *Ctr1* gene in GCs (*Ctr1*^*ΔGC*^) were generated. *Ctr1*^***Δ*GC**^ mice were examined at PND 14, 28 and 41. The *Ctr1*^***Δ*GC**^ mice were indistinguishable from their WT littermates with regards to their body weight and overall appearance. However, upon examining the testis, the *Ctr1*^***Δ*GC**^ mice at PND 28 and 41 had significantly reduced testicular weights as shown in Fig 2A. Similarly, significant differences in testis to body weight ratios were observed at PND 28 (a reduction of up to 60%) and PND 41 (up to 80% reduction) as compared to their WT littermates (Fig 2B). Fig 2C illustrates a marked reduction in the size of the testis of *Ctr1*^*ΔGC*^ versus WT at PND 41.

Histological evaluation of *Ctr1*^*ΔGC*^ mice testes revealed an increase in spermatogenic abnormalities with increasing age. At PND 14, WT and *Ctr1*^*ΔGC*^ testes appear similar; the first round of spermatogenesis has initiated and maturing spermatocytes (zygotene and pachytene) are present in the seminiferous tubules (Fig 2D and 2G, respectively). However, at PND 28, a degenerative seminiferous epithelium is evident in the *Ctr1*^*ΔGC*^ testes and various stages of GC subtypes were missing. In WT testis at PND 28 (Fig 2E), the most mature GC subtypes present in every seminiferous tubule were the haploid round spermatids along with elongated spermatids. Although round and elongated spermatids were present in 90% of tubules cross sections in *Ctr1*^*ΔGC*^ testis at PND 28, primary pachytene spermatocytes were either absent or present in low quantity (Fig 2H). Most of the residual GCs in PND 28 *Ctr1*^*ΔGC*^ testis had an abnormal appearance with condensed nuclei as well as SCs with large vacuoles (Fig 2H). By PND 41, when all subtypes of GCs are present in WT (Fig 2F), each tubule in the *Ctr1*^*ΔGC*^ testes were completely devoid of post-meiotic GCs with severe SC vacuolization (Fig 2I). However, the residual GCs that were present in the seminiferous tubules were mostly early stage primary spermatocytes (e.g. preleptotene and leptotene spermatocytes) and spermatogonial cells (Fig 2I). GCs beyond leptotene stage were absent in most tubules (i.e. pachytene spermatocytes and round spermatids).

Evaluation of the incidence of GC apoptosis did not show significant differences in the apoptotic index (AI) between the genotypes (*Ctr1*^*ΔGC*^ and WT) at PND 14. The *Ctr1*^*ΔGC*^ mice at PND 28 however, displayed a surge in apoptotic GCs, with ~35% of the tubules each containing >3 TUNEL positive GCs (Fig 2J). Apoptotic GCs were found both GCs around the periphery of the tubules and surrounding the lumen (Fig 2K). By PND 41, the AI was effectively zero in the *Ctr1*^*ΔGC*^ testis, due to the near-complete loss of GCs from the seminiferous epithelium.

### *Ctr1*^*ΔGC*^ testes at PND 41 contain undifferentiated spermatogonial cells with normal Sertoli cell number

**Fig 3:**
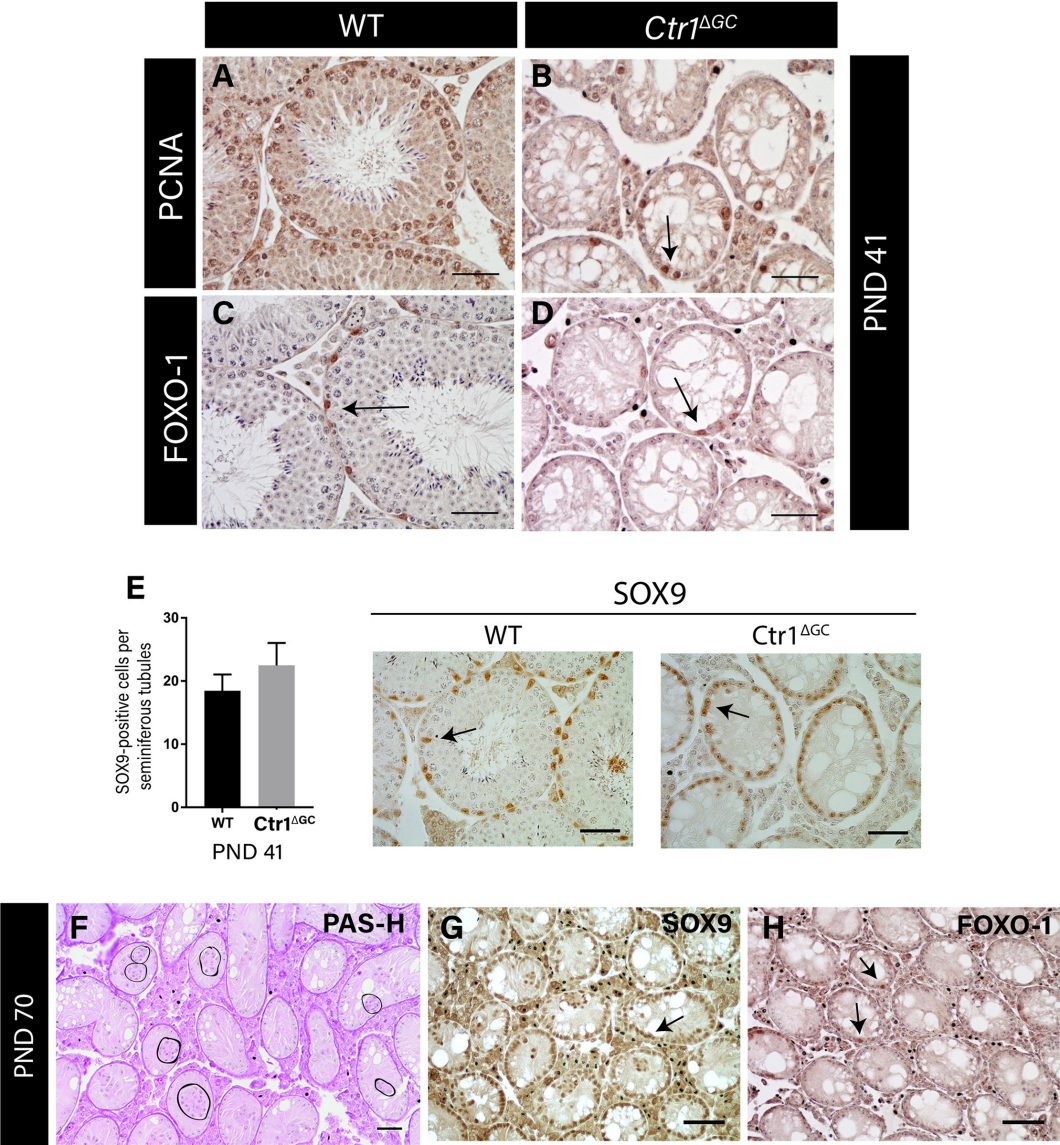
*Ctr1*^*ΔGC*^ mice at PND 41 display presence of undifferentiated spermatogonial cells and normal number of Sertoli cells in adult testes of Ctr1^*Δ*GC^ mice. Immunohistochemical nuclear staining for PCNA in GCs of both (A) WT and (B) *Ctr1*^*ΔGC*^ testes. Presence of FOXO-1 expressing undifferentiated spermatogonial cells at PND 41 in (C) WT and (D) *Ctr1*^*ΔGC*^ testes. Average number of SOX9-positive cells per seminiferous tubules (E). On right, representative immunohistochemical staining from SOX9 analysis of WT and *Ctr1*^*ΔGC*^; arrows indicating SOX9-positive SCs (Average ±SD, N = 3 for each genotype). Scale bar = 100 μm. Histological analysis on *Ctr1*^*ΔGC*^ testis at PND 70 indicating multiple clusters of SCs in the lumen indicated by circles (F) with PAS-H staining. SOX9-positive SCs clarifying the SCs clusters in the lumen (G). (H) Presence of FOXO-1-positive spermatogonial cells, indicated by arrows, within the tubules of *Ctr1*^*ΔGC*^ testis at PND 70. Scale = 50 μm.

Further analysis was performed to determine whether the residual GCs in *Ctr1*^*ΔGC*^ testes at PND 41 possess proliferative activity. The mitotic activity of GCs was analyzed based on nuclear proliferating cell nuclear antigen (PCNA) staining which marks cells that are in G1 and S phase of the cell cycle and is expressed in subsets of proliferative spermatogonia and in preleptotene through pachytene spermatocytes [36]. The presence of PCNA-positive GCs in the *Ctr1*^*ΔGC*^ testes at PND 41 (Fig 3B) indicates that the GCs have maintained their mitotic activity. The overall reduced PCNA-positive cells in *Ctr1*^*ΔGC*^ testis indicates the absence of leptotene and pachytene spermatocytes in *Ctr1*^*ΔGC*^ as compared to WT, shown in Fig 3A and 3B.

The severe loss of GCs by PND 41 in *Ctr1*^*ΔGC*^ testes indicates the loss of spermatogonial stem cells (SSC). To address this possibility, we assessed the expression of transcription factor, Forkhead box protein-O1 (FOXO-1), which is involved in SSC self-renewal and expressed by undifferentiated spermatogonial cells (A_s_, A_pr_ and A_al_) [37,38]. Since these cells continually arise from SSCs, they were used as an indirect indicator of the presence of the SSC pool. Fig 3C and 3D indicates the presence of FOXO1-positive cells with in the seminiferous tubules cross sections in *Ctr1*^*ΔGC*^ and WT testis at PND 41. Quantification of FOXO1-positive cells per tubule revealed no difference between *Ctr1*^*ΔGC*^ and WT testes at PND 41 (S2 Fig). To evaluate whether the numbers of SCs were altered in the testes of Ctr1^*Δ*GC^ mice, the numbers of cells staining positive for the SC specific marker SOX9 were evaluated. No significant changes in number of SCs per tubule were observed between the *Ctr1*^*ΔGC*^ and WT testes at PND 41 (Fig 3E).

Preliminary histological evaluation on *Ctr1*^*ΔGC*^ mice testes at PND 70 indicated a lack of regeneration of spermatogenesis, as shown in Fig 3F. Seminiferous tubules of *Ctr1*^*ΔGC*^ testis displayed clusters of SCs that protruded into the lumen, a symptom of prolonged SC inactivity due to loss of GCs found in testes of sterile mice [39] (Fig 3F and 3G). However, similar to *Ctr1*^*ΔGC*^ testis observed at PND 41, the *Ctr1*^*ΔGC*^ testes at PND 70 did contain FOXO-1 positive spermatogonia (Fig 3H).

### *Ctr1*^*ΔSC*^ mice at PND 60 are indistinguishable from WT littermates

**Fig 4:**
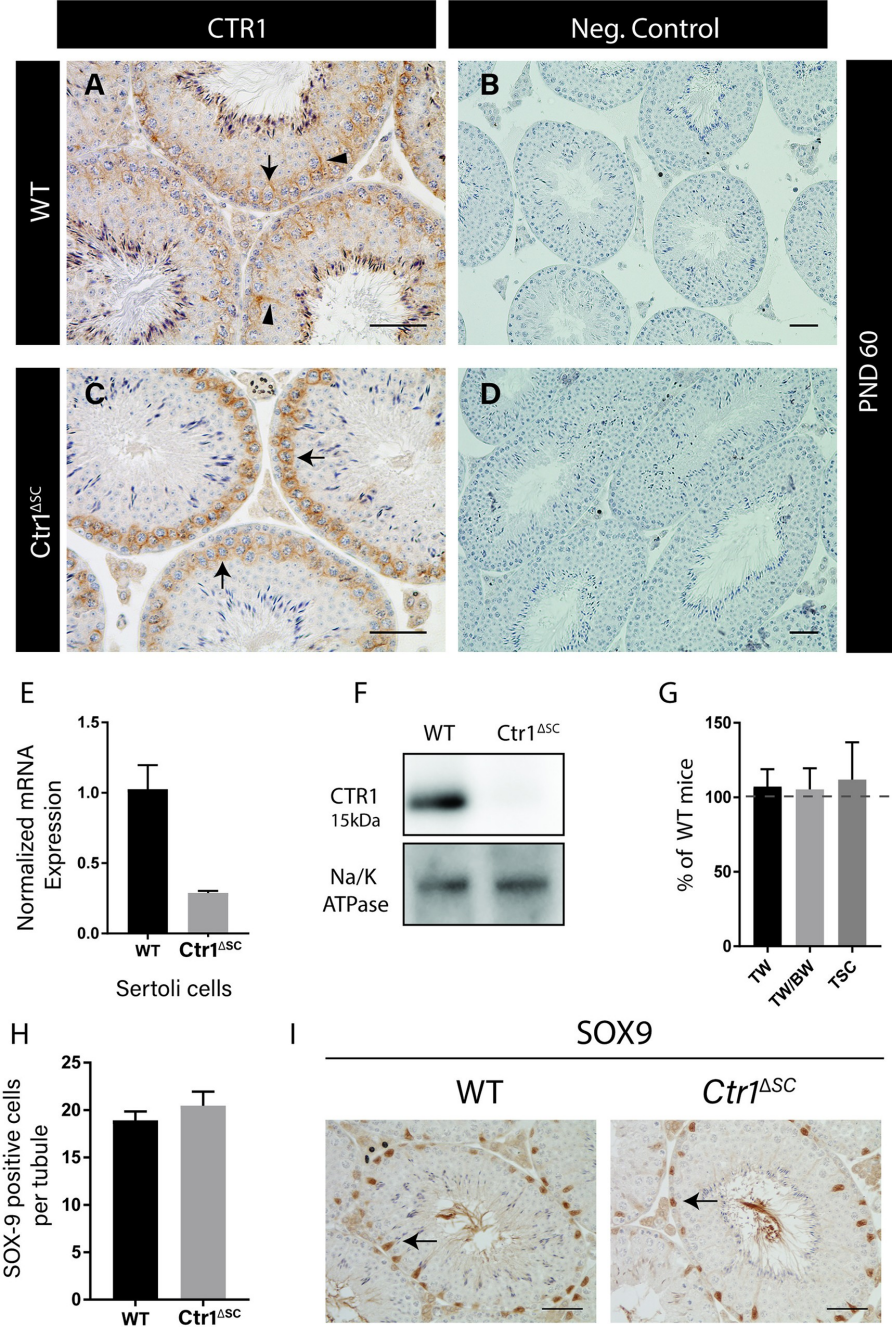
*Ctr1*^*ΔSC*^ mice at PND 60 are indistinguishable from WT littermates. Immunohistochemical analysis of CTR1 protein expression within the *Ctr1*^*ΔSC*^ and WT testis. (A) CTR1 localization on within the seminiferous tubules in WT mice expressed on the SCs (arrowheads) and spermatocytes (arrow), and CTR1 localization on the spermatocytes only (arrows) in the *Ctr1*^*ΔSC*^ testis (C). Negative secondary only control of WT and *Ctr1*^*ΔSC*^ testis shown on the right (B and D, respectively). (E) Q-PCR on *Ctr1* mRNA expression normalized to *Hprt1* gene indicates up to 70% reduced expression in *Ctr1*^*ΔSC*^ SCs compared to WT (average ± SEM). (F) Immunoblot analysis on crude membrane extract on WT and *Ctr1*^*ΔSC*^ SC isolates against CTR1 antibody shows undetectable CTR1 expression in *Ctr1*^*ΔSC*^ compared to WT mice. (G) Testicular weight (TW), testes to body weight ratio (TW/BW) and testicular spermatid head counts (TSC) all displayed similar to their WT littermates. The graph represents relative measurements of *Ctr1*^*ΔSC*^ mice compared to WT mice, dashed line represents 100% of each measurement in WT mice (average ± SD). (H) SOX9-positive cells within the seminiferous tubules of both WT and *Ctr1*^*ΔSC*^ testes displayed similar number of SCs. (I) Representative images of SOX9 staining indicated by arrows in both WT and *Ctr1*^*ΔSC*^ testis. Each data point is representative of 4–5 mice of each genotype. Scale bar = 100 μm.

To examine whether loss of CTR1 expression in SC would also affect spermatogenesis, mice with a specific disruption of the *Ctr1* gene in SC (*Ctr1*^*ΔSC*^) were generated. The specific knockout of *Ctr1* gene by the SCs was verified by immunohistochemical staining in testis cross sections of WT and *Ctr1*^*ΔSC*^ mice. Fig 4A indicates the localization of CTR1 on both on primary spermatocytes and on SCs in the WT testis, whereas CTR1 is localized only on primary spermatocytes within the seminiferous tubules of the *Ctr1*^*ΔSC*^ testis (Fig 4C). To further verify the knockout of *Ctr1* in SCs, *Ctr1* gene and protein expression of isolated SCs from both WT and *Ctr1*^*ΔSC*^ mice were compared using q-PCR and immunoblotting. Fig 4E indicates a 70% reduced in *Ctr1* mRNA expression by *Ctr1*^*ΔSC*^ SCs compared to WT SCs. Protein expression of CTR1 in crude membrane extracts of isolated SCs from *Ctr1*^*ΔSC*^ mice was undetectable compared to WT mice (Fig 4F). Similarly, testis histology of *Ctr1*^*ΔSC*^ mice at earlier age (PND 14) was observed to be comparable to their WT littermates (S3A and S3B Fig).

The *Ctr1*^*ΔSC*^ mice displayed similar appearance as their WT littermates; no obvious behavioral deficit was noted. Testes weight, testes to body weight ratio, and testicular spermatid head counts of both *Ctr1*^*ΔSC*^ and WT mice were comparable at PND 60 as indicated in Fig 4G. The *Ctr1*^*ΔSC*^ had similar numbers of SCs as their WT littermates shown in Fig 4H and 4I. When *Ctr1*^*ΔSC*^ males were crossed with WT females to assess fertility, normal length of conception and number of pups was observed in *Ctr1*^*ΔSC*^ male mice (average litter size 9 ± 1) compared to WT male mice (average litter size 9).

### *Ctr1*^*ΔSC*^ mice exhibit testicular copper deficiency

**Fig 5:**
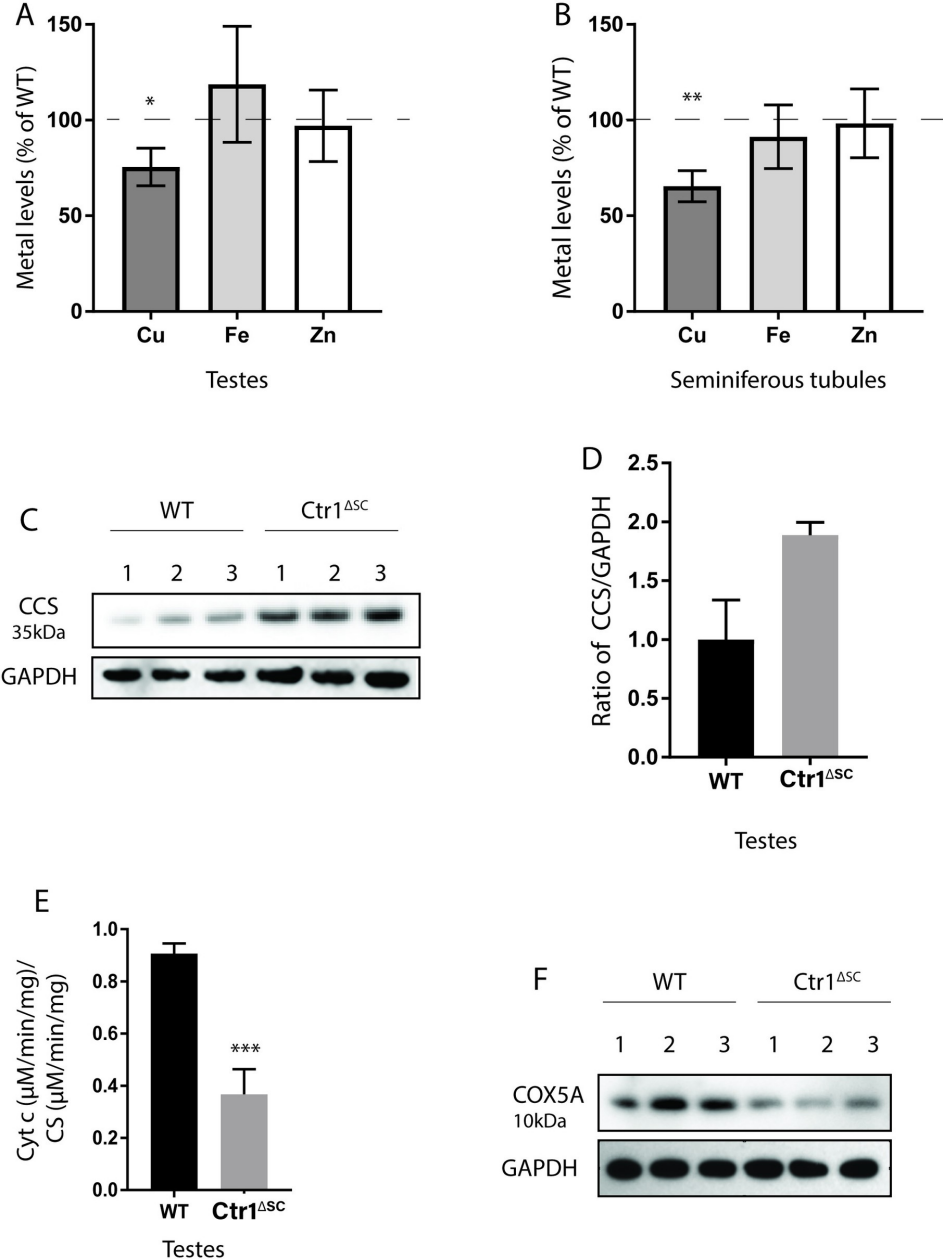
*Ctr1*^*ΔSC*^ mice exhibit testicular copper deficiency. **(**A) Relative Cu, Fe and Zn levels in *Ctr1*^*ΔSC*^ testis and in (B) *Ctr1*^*ΔSC*^ seminiferous tubules compared to WT testis. Data represent percent concentration (μg/g dry tissue weight) of *Ctr1*^*ΔSC*^ tissues compared to WT tissues (average ±SD). Dashed line represents 100% of each metal in WT tissues. For each genotype N = 4–5, *P<0.05, **p<0.005. (C) Immunoblot analysis on CCS and GAPDH is shown as loading control. Three animals were randomly selected and analyzed from each WT and *Ctr1*^*ΔSC*^ mice. Each number above represents an animal from each genotype. (D) Quantified protein expression of CCS normalized to GAPDH expression (average ± SEM, N = 3, p = 0.0656). (E) Cytochrome c oxidase (CCO) activity of WT and *Ctr1*^*ΔSC*^ testis. Tissue lysates from randomly selected three WT and *Ctr1*^*ΔSC*^ mice were analyzed to measure CCO activity. Graph represents the rate of cytochrome c (cyt c) oxidation (μM/min/mg of protein lysate) of *Ctr1*^*ΔSC*^ and WT testis and normalized to citrate synthase (CS) activity for each sample (average ± SD, N = 3, ***p<0.001). (F) Immunoblot analysis on the COX5A, a CCO complex subunit, and GAPDH is shown as loading control.

Since *Ctr1*^*ΔSC*^ mice exhibited normal fertility, further experiments were carried out to examine whether CTR1 in SCs is involved in Cu homeostasis in the testis. The Cu concentration of *Ctr1*^*ΔSC*^ mice testis displayed up to 30% reduced steady-state Cu levels compared to their WT littermates (Fig 5A). The Cu levels were further analyzed within the seminiferous tubules, which include both GCs and SCs. The *Ctr1*^*ΔSC*^ mice displayed up to 40% reduced Cu levels compared to WT as shown in Fig 5B. To examine whether reductions in the steady-state levels of Cu had any effect on other metals, iron and zinc were evaluated in testes and seminiferous tubules, but no differences were observed (Fig 5A and 5B).

Based on reduced testicular steady-state Cu levels in *Ctr1*^*ΔSC*^ mice, we examined whether these changes affected Cu-dependent enzyme activities or cellular regulations. Cu-chaperone for superoxide dismutase (CCS) protein captures and supplies Cu to the Cu, Zn-superoxide dismutase (SOD1) enzyme and reduction in Cu levels have been shown to cause up-regulation of CCS, hence CCS protein levels are inversely proportional to their intracellular Cu bioavailability [40]. Indeed, *Ctr1*^*ΔSC*^ testes exhibited elevated (p = 0.0656) protein level of steady-state CCS compared to WT, indicating testicular Cu deficiency (Fig 5C and 5D). Further analysis was performed on the cuproenzyme cytochrome c oxidase (CCO), which is the fourth complex of the respiratory chain reaction within the mitochondria that requires Cu for the assembly of the subunits within complex 4 [41]. The CCO activity and protein expression of the testis from both genotypes were assessed. As predicted, *Ctr1*^*ΔSC*^ testes exhibited up to 60% reduction in CCO activity compared to WT testes (Fig 5E). Consistent with the reduced CCO activity, COX5A, a CCO complex subunit, protein levels were also reduced in *Ctr1*^*ΔSC*^ testes as compared to WT testes, as shown in Fig 5F.

### Loss of CTR1 in Sertoli cells display normal copper levels but copper deficient phenotype

**Fig 6:**
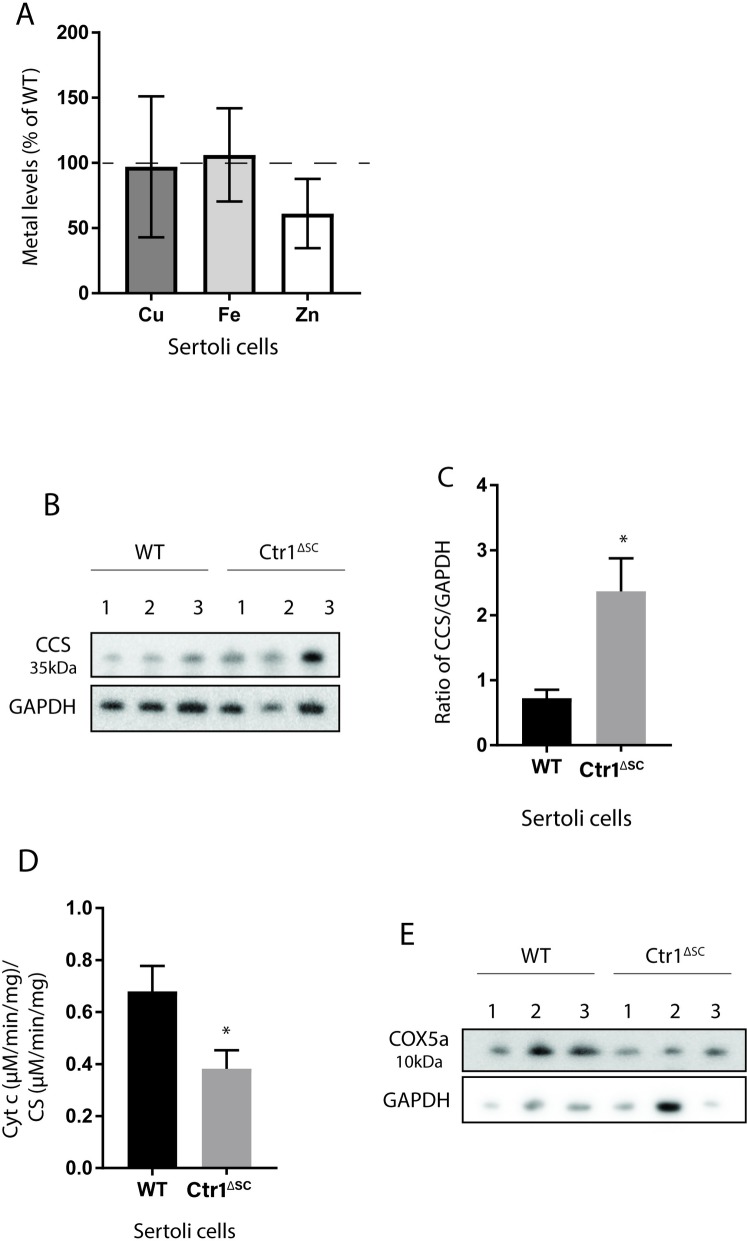
Loss of CTR1 in Sertoli cells display normal copper levels but copper deficient phenotype. (A) Relative Cu, Fe and Zn levels in primary SCs isolated from WT and *Ctr1*^*ΔSC*^ testes. Data represents percent concentration (μg of metal per 10,000 cells) of *Ctr1*^*ΔSC*^ SCs compared to WT SCs. Dashed line represents 100% of each metal in WT SCs. (average ± SD, N = 5–7). (B) Immunoblot analysis (C) quantified protein expression of CCS normalized to GAPDH expression. Primary SCs isolated from three animals analyzed from each WT and *Ctr1*^*ΔSC*^ mice (average ± SEM, N = 3, *p<0.05). (D) CCO activity of WT and *Ctr1*^*ΔSC*^ primary SCs. Cell lysates from three different WT and *Ctr1*^*ΔSC*^ mice were analyzed to measure CCO activity. Graph represents the rate of cyt c oxidation (μM/min/mg of protein lysate) of WT and *Ctr1*^*ΔSC*^ SCs and normalized CS activity for each sample (average ± SD, N = 3, *p<0.05). (E) Immunoblot analysis on the COX5A expression of primary SCs derived from three individual mice from each genotype. GAPDH is shown as loading control.

Since whole testis and seminiferous tubules displayed a Cu deficient status, we further examined Cu levels in primary SCs derived from immature (PND 25) *Ctr1*^*ΔSC*^ and WT mice testes. Intracellular Cu levels was measured using ICP-MS on primary SC of WT and *Ctr1*^*ΔSC*^ mice. Surprisingly, Cu levels in *Ctr1*^*ΔSC*^ SCs were similar to Cu levels in WT SCs as indicated in Fig 6A. However, SCs of *Ctr1*^*ΔSC*^ mice displayed almost two-fold increase in CCS protein levels when compared to SCs of WT mice as shown in Fig 6B and 6C. Similarly, SCs of *Ctr1*^*ΔSC*^ mice exhibited a 45% reduced CCO enzyme activity and reduced protein expression of COX5A as compared to SCs of WT mice (Fig 6D and 6E) indicating lower Cu bioavailability as compared to the WT SCs even though intracellular Cu levels were similar to WT.

## Discussion

The expression pattern of the *Ctr1* gene and protein has been demonstrated in mice testis, but the physiological significance of CTR1 localization within the cell types of seminiferous tubules has not been identified [15,22]. In order to interrogate and differentiate the physiological significance of CTR1 protein in functional spermatogenesis, we developed and characterized both GC- specific (*Ctr1*^*ΔGC*^) and SC-specific (*Ctr1*^*ΔSC*^) *Ctr1* knockout mice.

Primary spermatocytes are in meiotic prophase I which go through series of morphological transition phases, starting from pre-leptotene, leptotene, zygotene, pachytene, and diplotene spermatocytes [9]. The high localization of CTR1 on pachytene spermatocytes implies that precise levels of Cu are needed for spermatocytes to progress through meiotic prophase I, as observed in yeast and *Drosophila* models [19,21]. The reason why CTR1 is specifically expressed at pachytene stage is not clear. However, it may reflect the high energy demand and mitochondrial biogenesis that occurs during meiosis. Mammalian testes have a series of testis-specific mitochondrial protein subunits that are expressed in GCs [42,43]. Cytochrome-c oxidase (CCO) is ubiquitously expressed, however in mammalian testes, the CCO subunit II, which contains the Cu binuclear binding sites, are highly expressed in pachytene spermatocytes [44]. Interestingly, a testis-specific cytochrome c (cyt c_t_) is predominantly expressed in zygotene and pachytene spermatocytes which is believed to have a distinct role in GC mitochondria that is different from that of somatic cyt c in that it interacts with the testis-specific CCO enzyme [43–46]. Therefore, the expression and regulation of the cell type-specific mitochondrial proteins may underscore the specific localization of CTR1 on pachytene spermatocytes.

Using the *Ctr1*^*ΔGC*^ mice model, we demonstrated physiological evidence that CTR1 is required in GCs for the establishment of functional spermatogenesis during puberty (PND28). At PND 14 the *Ctr1*^*ΔGC*^ testis displays a phenotype indistinguishable from that of the WT littermates with the presence of early stages of primary spermatocytes [9]. In mice, the process of a SSCs to produce elongated spermatids take 35 days [9,47]. At PND 28, the presence of round spermatids, but lack of primary pachytene spermatocytes in *Ctr1*^*ΔGC*^ tubules suggest that the first round of spermatogenesis has occurred leading to production of spermatids, but the subsequent cycle has failed to complete; resulting in seminiferous tubules that are almost devoid of later stages of spermatocytes and spermatids by PND 41 and in older mice (PND70). The presence of early spermatocytes (preleptotene and leptotene), PCNA- and FOXO-1-positive cells in most tubule cross section of the *Ctr1*^*ΔGC*^ testis indicate that the proliferative and undifferentiated spermatogonia are still being generated from SSC pool, however the progression of spermatogenesis beyond leptotene stage of GC development is lost. In align with what previously discussed, Nakada et. al. group observed similar phenotype in adult mice (PND >100) with severe mitochondrial respiratory defects exhibit meiotic arrest at zygotene stage and enhanced apoptosis. Although our analysis on spermatocyte subtypes is based on histological assessment of the seminiferous tubule cross sections, further and more detailed analysis on spermatocyte stages can illustrate whether CTR1-dependent Cu import in GCs is essential for mitochondrial respiratory activity for the progression to the pachytene stages during meiosis.

These observations, along with the stage-specific expression of CTR1 on pachytene spermatocytes support the notion that the spermatogenic defect occurs after the first round of spermatogenesis and arrests prior to pachytene stage of meiosis. Why the early phase of first round of spermatogenesis appears to occur normally remains unclear, but could indicate that the requirement for Cu transport is age dependent. In support of this notion, testicular concentration of Cu varies with age; male mice have low levels at PND ~7 which increases to peak levels by PND 20, and then declines through PND ~180 [22,48]. Whether Cu supplementation can rescue the *Ctr1*^*ΔGC*^ mice phenotype requires further investigation which would determine if the spermatogenic failure is due to the age specific role of CTR1 in Cu absorption or perhaps due to a yet appreciated function of the CTR1 protein in GC development in mice. Given the conserved function of Ctr1 in eukaryotes, Streiger et al reported that the male sterility with loss of Ctr1C was rescued with Cu supplementation. The presence of spermatogonial cells in adult *Ctr1*^*ΔGC*^ mice provides the possibility of spermatogenic recovery in *Ctr1*^*ΔGC*^ mice by Cu supplementation to be plausible.

Functional spermatogenesis is highly dependent on the support system of the neighboring somatic SCs. SCs are polarized epithelial cells that function to ensure a suitable microenvironment for spermatogenesis to take place by regulating the secretion and transportation of essential ions and metabolites to the developing GCs [10]. As we have demonstrated, CTR1 is predominantly localized on the primary pachytene spermatocytes which are located in the adluminal compartment within the BTB. This observation suggested that the source of Cu in the adluminal compartment may be, in part, from the SCs, and that the CTR1 expression by SCs may play a role in functional spermatogenesis. To test this hypothesis, *Ctr1*^*ΔSC*^ mice were generated. Unexpectedly, *Ctr1*^*ΔSC*^ mice testes exhibited comparable spermatogenesis and fertility as their WT littermates suggesting that the CTR1 expression in SCs is dispensable for normal spermatogenesis to occur. However, the overall reduced Cu levels and Cu-dependent protein activity and expression in both the whole testis and in the seminiferous tubules, suggests that CTR1 in SCs does function, at least, partly in Cu transport in testis. Given that deletion of *Ctr1* in GCs manifest defects in GC development, the importance of Cu delivery for GCs may have exerted the SCs to develop compensatory or a separate Cu acquisition pathway to provide essential supply of Cu for the GCs. This is evident in that only a 30% reduction in Cu level and 60% reduction in CCO activity were observed in the testis of *Ctr1*^*ΔSC*^ mice, and that *Ctr1*^*ΔSC*^ mice were able to sire pups. Normal spermatogenesis observed in *Ctr1*^*ΔSC*^ mice despite the reduced testicular Cu level contradicts with previous reports that have documented impairment of testicular function in Cu deficient animals [6–8]. Although the testicular Cu level was not stated in these studies, this discrepancy can be due to the systemic Cu deficiency in animals rather than tissue/cell specific Cu deficiency. Our analysis on the fertility of the *Ctr1*^*ΔSC*^ mice is limited on testicular spermatid head count thus we cannot rule out the possibility that loss of CTR1 in SCs affects sperm quality in these mice as a secondary effect. Nevertheless, *Ctr1*^*ΔSC*^ mice sired comparable number of pups when mated with virgin WT females as to WT males.

Interestingly, *in vitro* analysis of intracellular Cu levels of primary SCs derived from WT and *Ctr1*^*ΔSC*^ mice displayed comparable levels of Cu even though SCs of *Ctr1*^*ΔSC*^ mice exhibited Cu deficient phenotype. CTR1 protein has been shown to localize both on plasma membrane and on intracellular vesicles, depending on the cell type [25,49]. It is demonstrated on previous reports that CTR1 is involved in both importing extracellular Cu, as well as importing Cu from endosomal compartment [49]. Therefore, these observations may suggest that perhaps CTR1 in SCs mainly functions in mobilizing Cu from intracellular vesicle, which could explain why the SCs in *Ctr1*^*ΔSC*^ display a deficiency in Cu that is biologically unavailable. The possibility of compensatory Cu acquisition mechanism affecting Cu levels in cultured primary SCs cannot be ignored. However, given that *Ctr1*^*ΔSC*^ testis had 30% reduction in Cu level, together suggest that there may be an alternative Cu acquisition system that partially compliments CTR1-dependent Cu uptake pathway. A second Cu transporter, CTR2 (SLC31A2), which is structurally related to CTR1 protein, has been reported to express high levels in testis [17]. CTR2 functions as a low affinity Cu importer, Cu exporter from intracellular vesicle to cytoplasm, and as a regulator of macropinocytosis, [50–52] and thus it could play a role in Cu acquisition with loss of CTR1 in SCs both *in vivo* and *in vitro*.

Reduced Cu levels in testis and in seminiferous tubules, but not in SCs, suggests the possibility that CTR1 in SCs functions in efficiently transporting or mobilizing Cu from basolateral compartment to the adluminal compartment of the SCs to supply the GCs, which can explain the stage specific localization of CTR1 in SCs along the basal and adluminal compartment of the seminiferous tubules. A similar observation was made in mice with intestinal epithelial cell-specific *Ctr1* knockout [25]. These mice exhibited Cu accumulation in enterocytes even though the intestinal epithelial cells itself and other peripheral organs displayed Cu deficiency, demonstrating another role of CTR1 in mobilizing Cu from the intracellular vesicle to other organelles and subsequently to the peripheral organs [17,25]. Therefore, it is possible that with loss of CTR1 in SCs, Cu is held in SCs that may not be biologically available and that Cu deficiency observed in testis and in seminiferous tubules may be primarily due to Cu deficiency in GCs. Alternatively, CTR1 in SCs may function in Cu uptake that is released from GCs, in order to protect GCs from Cu overload, or recycle Cu back to the primary spermatocytes, a pathway analogous to how iron is recycled and maintained within the seminiferous tubules [53]. However, given that the testis and seminiferous tubules of *Ctr1*^*ΔSC*^ mice exhibited significant Cu deficiency, may not support the latter theory.

In summary, we have demonstrated for the first time, the differential physiological significance of CTR1 in two different testicular cell types, GCs and SCs. The high expression of CTR1 on pachytene spermatocytes and the progressive failure of spermatogenesis in adult *Ctr1*^*ΔGC*^ mice, points to the critical requirement of CTR1 for mammalian spermatogenesis. In contrast, the loss of expression of CTR1 in SCs did not negatively affect spermatogenesis even though the testis of these mice exhibited a significant reduction in Cu. Taken together, these observations support the hypothesis that CTR1 protein expression is required for GC development and functional spermatogenesis.

## Supporting information

S1 Fig (A) Breeding strategies for generating GC specific (*Ddx4-Cre*) and SC specific (*Amh- Cre1*) knockout mice. A schematic depiction of alleles encoding for Ctr1 gene. White box represents Ctr1 structural gene, grey triangle represents loxP sites flanking the Ctr1 gene. Cre recombinase gene under the *Ddx4* or *Amh* or promoter gene is represented as black and white box, where white is Cre recombinase gene and black is promoter gene. Initially, homozygous *Ctr1fl/fl* female mice were crossed with male mice carrying either *Ddx4-* or *Amh-Cre* gene with WT *Ctr1+/+* genotype. The heterozygous male mice carrying either *Ddx4-Cre*; *Ctr1+/fl* or *Amh-Cre*; *Ctr1 +/fl* genotype from the initial cross were then back-crossed to *Ctr1fl/fl* female mice to obtain GC (*Ddx4- Cre;Ctr1 fl/Δ*, *Ctr1ΔGC)* or SC specific (*Amh-Cre;Ctr1 fl/Δ*, *Ctr1ΔSC) Ctr1* knockout mice. (B) Representative PCR genotyping results showing mouse tail DNA samples with WT (*Ctr1+/+*), heterzogous floxed (*Ctr1fl/+*), and homozygous floxed mouse (*Ctr1fl/fl*). (C) Immunohistochemical analysis of stage specific CTR1 protein expression in adult C57BL/6J testes cross section. Arrows indicates CTR1 on pachytene spermatocytes. Arrowheads indicate CTR1 on SCs.
(PDF)

S2 Fig Graph showing average number of FOXO1-positive cells per tubules (calculating based on the total number of tubules analyzed). For each animal/genotype ≥100 tubules were counted.
(PDF)

S3 Fig Histological cross section of WT (A) and Ctr1ΔSC (B) mice testis at PND 14. Scale = 180μm.
(PDF)
