# Supplementary figures and images for: Copper transporter 1 (CTR1) expression by mouse testicular germ cells, but not Sertoli cells, is essential for functional spermatogenesis
Source: PLoS One. 2019 Apr 19;14(4):e0215522. doi: 10.1371/journal.pone.0215522 (PMC6474593; doi:10.1371/journal.pone.0215522)

A

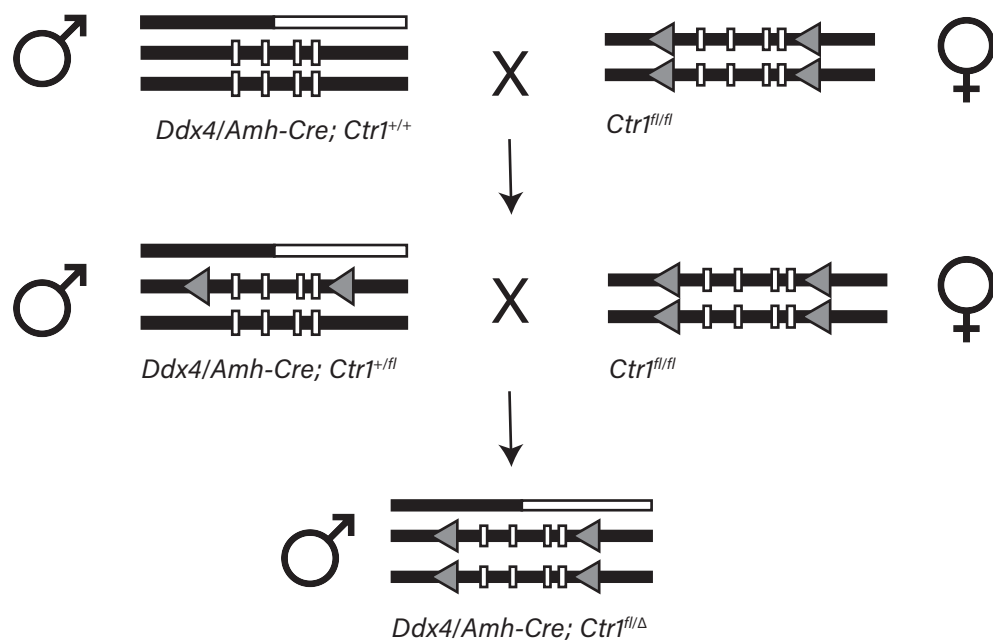

B

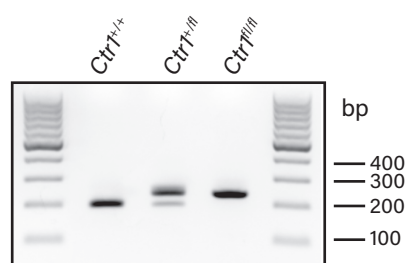

C

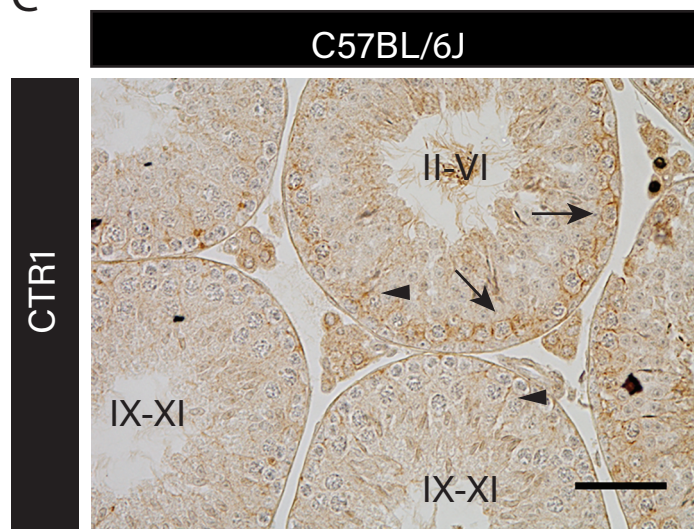

Supplement: S1 Fig — (A) Breeding strategies for generating GC specific (Ddx4-Cre) and SC specific (Amh- Cre1) knockout mice. A schematic depiction of alleles encoding for Ctr1 gene. White box represents Ctr1 structural gene, grey triangle represents loxP sites flanking the Ctr1 gene. Cre recombinase gene under the Ddx4 or Amh or promoter gene is represented as black and white box, where white is Cre recombinase gene and black is promoter gene. Initially, homozygous Ctr1fl/fl female mice were crossed with male mice carrying either Ddx4- or Amh-Cre gene with WT Ctr1+/+ genotype. The heterozygous male mice carrying either Ddx4-Cre; Ctr1+/fl or Amh-Cre; Ctr1 +/fl genotype from the initial cross were then back-crossed to Ctr1fl/fl female mice to obtain GC (Ddx4- Cre;Ctr1 fl/Δ, Ctr1ΔGC) or SC specific (Amh-Cre;Ctr1 fl/Δ, Ctr1ΔSC) Ctr1 knockout mice. (B) Representative PCR genotyping results showing mouse tail DNA samples with WT (Ctr1+/+), heterzogous floxed (Ctr1fl/+), and homozygous floxed mouse (Ctr1fl/fl). (C) Immunohistochemical analysis of stage specific CTR1 protein expression in adult C57BL/6J testes cross section. Arrows indicates CTR1 on pachytene spermatocytes. Arrowheads indicate CTR1 on SCs. (PDF) [file pone.0215522.s001.pdf]

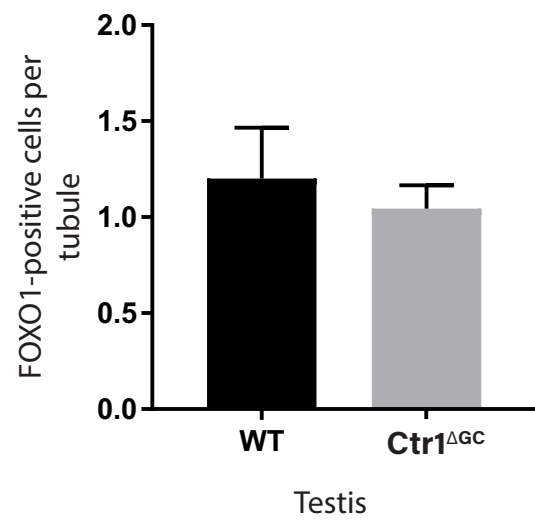

Supplement: S2 Fig — For each animal/genotype ≥100 tubules were counted. (PDF) [file pone.0215522.s002.pdf]

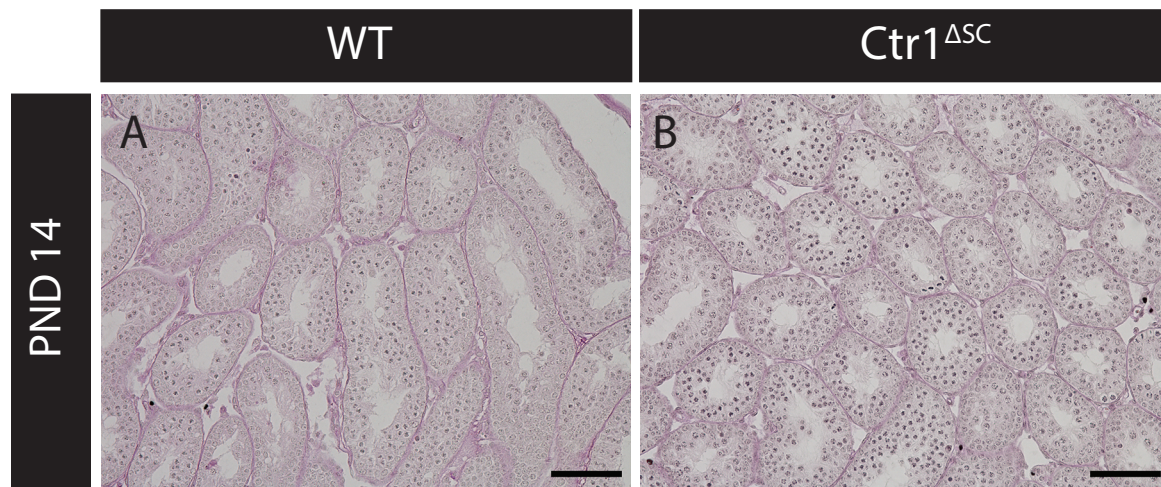

Supplement: S3 Fig — Histological cross section of WT (A) and Ctr1ΔSC (B) mice testis at PND 14. Scale = 180μm. (PDF) [file pone.0215522.s003.pdf]
